# SNP Discovery and Chromosome Anchoring Provide the First Physically-Anchored Hexaploid Oat Map and Reveal Synteny with Model Species

**DOI:** 10.1371/journal.pone.0058068

**Published:** 2013-03-22

**Authors:** Rebekah E. Oliver, Nicholas A. Tinker, Gerard R. Lazo, Shiaoman Chao, Eric N. Jellen, Martin L. Carson, Howard W. Rines, Donald E. Obert, Joseph D. Lutz, Irene Shackelford, Abraham B. Korol, Charlene P. Wight, Kyle M. Gardner, Jiro Hattori, Aaron D. Beattie, Åsmund Bjørnstad, J. Michael Bonman, Jean-Luc Jannink, Mark E. Sorrells, Gina L. Brown-Guedira, Jennifer W. Mitchell Fetch, Stephen A. Harrison, Catherine J. Howarth, Amir Ibrahim, Frederic L. Kolb, Michael S. McMullen, J. Paul Murphy, Herbert W. Ohm, Brian G. Rossnagel, Weikai Yan, Kelci J. Miclaus, Jordan Hiller, Peter J. Maughan, Rachel R. Redman Hulse, Joseph M. Anderson, Emir Islamovic, Eric W. Jackson

**Affiliations:** 1 General Mills Crop Biosciences, Kannapolis, North Carolina, United States of America; 2 Eastern Cereal and Oilseed Research Centre, Agriculture and Agri-Food Canada, Ottawa, Ontario, Canada; 3 Western Regional Research Center, Genomics and Gene Discovery, United States Department of Agriculture - Agricultural Research Service, Albany, California, United States of America; 4 Biosciences Research Lab, United States Department of Agriculture - Agricultural Research Service, Fargo, North Dakota, United States of America; 5 Department of Plant and Wildlife Sciences, Brigham Young University, Provo, Utah, United States of America; 6 Cereal Disease Laboratory, United States Department of Agriculture - Agricultural Research Service, Saint Paul, Minnesota, United States of America; 7 Department of Agronomy and Plant Genetics, University of Minnesota, Saint Paul, Minnesota, United States of America; 8 Limagrain Cereal Seeds, Lafayette, Indiana, United States of America; 9 Small Grains and Potato Germplasm Research Unit, United States Department of Agriculture - Agricultural Research Service, Aberdeen, Idaho, United States of America; 10 Department of Evolutionary and Environmental Biology and Institute of Evolution, University of Haifa, Haifa, Israel; 11 Crop Development Centre, Department of Plant Sciences, University of Saskatchewan, Saskatoon, Saskatchewan, Canada; 12 Department of Plant and Environmental Sciences, Norwegian University of Life Sciences, Ås, Norway; 13 Robert W. Holley Center for Agriculture and Health, United States Department of Agriculture - Agricultural Research Service, Ithaca, New York, United States of America; 14 Department of Plant Breeding and Genetics, Cornell University, Ithaca, New York, United States of America; 15 Eastern Regional Small Grains Genotyping Laboratory, North Carolina State University, United States Department of Agriculture - Agricultural Research Service, Raleigh, North Carolina, United States of America; 16 Cereal Research Centre, Agriculture and Agri-Food Canada, Winnipeg, Manitoba, Canada; 17 School of Plant, Environmental and Soil Sciences, Louisiana State University, Baton Rouge, Louisiana, United States of America; 18 Institute of Biological, Environmental and Rural Sciences, Aberystwyth University, Aberystwyth, Ceredigion, United Kingdom; 19 Department of Soil and Crop Sciences, Texas A&M University, College Station, Texas, United States of America; 20 Department of Crop Sciences, University of Illinois at Urbana-Champaign, Urbana, Illinois, United States of America; 21 Department of Plant Sciences, North Dakota State University, Fargo, North Dakota, United States of America; 22 Department of Crop Science, North Carolina State University, Raleigh, North Carolina, United States of America; 23 Department of Agronomy, Purdue University, West Lafayette, Indiana, United States of America; 24 JMP, SAS Institute Incorporated, Cary, North Carolina, United States of America; China Agricultural University, China

## Abstract

A physically anchored consensus map is foundational to modern genomics research; however, construction of such a map in oat (*Avena sativa* L., 2*n* = 6*x* = 42) has been hindered by the size and complexity of the genome, the scarcity of robust molecular markers, and the lack of aneuploid stocks. Resources developed in this study include a modified SNP discovery method for complex genomes, a diverse set of oat SNP markers, and a novel chromosome-deficient SNP anchoring strategy. These resources were applied to build the first complete, physically-anchored consensus map of hexaploid oat. Approximately 11,000 high-confidence *in silico* SNPs were discovered based on nine million inter-varietal sequence reads of genomic and cDNA origin. GoldenGate genotyping of 3,072 SNP assays yielded 1,311 robust markers, of which 985 were mapped in 390 recombinant-inbred lines from six bi-parental mapping populations ranging in size from 49 to 97 progeny. The consensus map included 985 SNPs and 68 previously-published markers, resolving 21 linkage groups with a total map distance of 1,838.8 cM. Consensus linkage groups were assigned to 21 chromosomes using SNP deletion analysis of chromosome-deficient monosomic hybrid stocks. Alignments with sequenced genomes of rice and *Brachypodium* provide evidence for extensive conservation of genomic regions, and renewed encouragement for orthology-based genomic discovery in this important hexaploid species. These results also provide a framework for high-resolution genetic analysis in oat, and a model for marker development and map construction in other species with complex genomes and limited resources.

## Introduction

Cultivated hexaploid oat (*Avena sativa* L.; 2*n* = 6*x* = 42, AACCDD) is a nutritionally important cereal crop [1] produced for both food and animal feed in many parts of the world. Plant breeders in many countries strive to develop improved oat varieties that incorporate better agronomic and nutritional traits as well as improved suitability for milling and processing. This work would be facilitated by a better framework of genetic and genomic information that can be used to enhance germplasm improvement in this crop. Unfortunately, genomic knowledge and resources in oat have lagged behind those in some other crop species despite a long history of research in this important crop. Previously-published hexaploid oat maps contain more than the predicted 21 linkage groups, and alignment among maps has been fragmentary [2], [3]. Difficulties have included a lack of sequence data, the large size and complexity of the genome [4], and instability of aneuploid mapping stocks. Unlike other common polyploids, colinearity among oat subgenomes is disrupted by numerous chromosomal rearrangements [5]–[9], thus diploid relatives have provided limited guidance for map construction. Prior genotyping in oat has relied heavily on DNA manipulation, hybridization, and size-discrimination [10]–[12], with heterogeneous results that are poorly integrated among different mapping populations. This problem has been confounded by sequence redundancy among polyploid sub-genomes, causing duplicate marker loci [13], [14].

Recently, a pilot study has demonstrated the potential to overcome some of these issues through discovery and mapping of highly-filtered SNP markers [15]. Transcriptome data linked to physically anchored genetic maps can facilitate genomic research and crop improvement, especially in plant species without sequenced genomes [16], [17]. Development of this resource in orphaned crops has become possible through affordable sequencing technologies and high-throughput genotyping platforms [18], [19]. These resources could enable development of the first physically-anchored consensus map in hexaploid oat.

Anchoring of a transcriptome-based genetic map to chromosomes provides validation of linkage groups, integration of genetic and cytogenetic data, and a foundation for genome sequencing and comparative genomics. However, this too has been a challenge in oat. Chromosome-deficient cytogenetic stocks have been used to assign molecular markers to corresponding chromosomes [20], [21], but such stocks are limited and require frequent monitoring to detect univalent shifts and disomic reversion [22], [23]. A partial oat monosomic series has been developed [23] and used to assign 22 linkage groups to 16 chromosomes [24]. However, this technique did not directly interrogate the F_1_ plant and did not account for cytogenetic variations, two factors which limit resolution.

The objectives of this current work were to develop, in hexaploid oat: (i) robust SNP assays; (ii) a new chromosome anchoring strategy; (iii) the first physically-anchored consensus map; and (iv) a comprehensive orthology-based comparison to model grass genomes. These results open a new window of scientific opportunity to explore other complex genomes, and will accelerate the genetic improvement of oat, an important functional food [1].

## Results

### *In Silico* SNP Discovery

**Figure 1:**
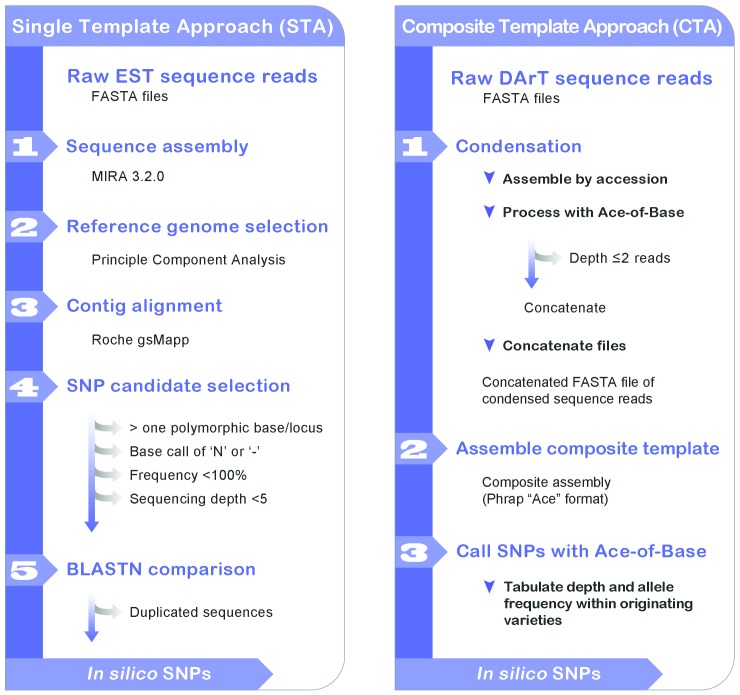
*In silico* SNP discovery approaches. Both methods started with a set of quality-trimmed 454 sequence reads identified by source germplasm from either the cDNA libraries or from DArT-based genomic complexity reductions. A. In the single template approach (STA), the reads were assembled by MIRA software to generate a consensus sequence for each contig. Four reference genomes were selected based on membership in four different quadrants of a principle component analysis that had been conducted previously using DArT markers. Consensus sequences from all varieties were assembled against each of the four reference genomes, and candidate SNPs were called using Roche GSMapper. SNPs were filtered based on several criteria, as described in the methods. Redundant SNPs were identified using BLASTN. B. In the composite template approach (CTA), reads were assembled within varieties at a high stringency using DNAstar Seqman Software. Then the consensus reads were filtered and truncated to include only those parts having perfect alignment with greater than two reads. The consensus reads were then concatenated and subjected to a single composite assembly at lower stringency. The consensus from this assembly was used as a composite reference genome to call SNPs. Although the SNP calling and filtering process was similar to CTA, this pipeline was automated using in-house software called “Ace-of-Base”.

More than 35 million un-filtered SNPs were predicted from cDNA reads using the single-template approach (STA) (Fig. 1). Stringent filtering based on insufficient read depth (<5 reads), heterogeneity within a variety, insertion/deletion polymorphism, or an ambiguous reference base left 75,974 candidate SNPs. Remaining SNPs were filtered by Illumina design scores (>0.8) and redundancy, then sorted in descending order by predicted minor-allele frequency (MAF), as estimated from the number of varieties that differed from the allele in the reference genome. For example, if 6 varieties differed out of a possible 20, the MAF was predicted as 30%. The top 2,270 cDNA-based SNP predictions were incorporated into pilot assays (Table 1).

**Table 1:** Summary of SNPs by marker discovery method.

Discovery method*	Prefix	No. tested	Total good	Conversion rate (%)	No. SNPs Mapped	%	No. calls Diversity	%
cDNA – STA	GMI_ES01-17	2270	991	44%	757	33%	878	39%
cDNA – CTA	GMI_ES_CC	336	144	43%	98	29%	133	40%
DArT – CTA	GMI_DS_CC	300	121	40%	87	29%	108	36%
DArT – Sanger	GMI_DS_A, oPt	66	48	73%	36	55%	43	65%
Genomic Tetraploid	GMI_grs	100	7	7%	7	7%	7	7%
**Totals**		**3072**	**1311**	**44%**	**985**	**32%**	**1169**	**38%**

* SNP discovery methods are based on SNP calls using an assembly against a template made of contigs from a single variety (STA), contigs assembled from multiple varieties (CTA), or Sanger sequences from DArT clones (Sanger).

The composite-template approach (CTA) utilized a multi-step procedure (Fig. 1): assembling within varieties, condensing to regions represented by three or more reads, then reassembling across varieties to predict 18,396 cDNA templates and 12,180 DArT templates with average lengths of 560 and 300 bp, respectively. Condensation reduced the computational load for the composite assembly and diminished the impact of read-errors. After re-assembling condensed reads against composite templates, 126,235 cDNA and 53,974 DArT SNPs were predicted. Filtering was applied to eliminate SNPs that were heterogeneous within varieties, had less than 10% MAF, or had fewer than 50 non-variable bases on either side. This resulted in a highly enriched candidate set of 1,056 cDNA SNPs and 519 DArT SNPs. Based on MAF, the top 336 cDNA-based SNPs and 300 DArT SNPs non-redundant with the STA were selected for validation (Table 1). An additional 66 sequences selected by manual inspection of Sanger sequences, and 100 SNPs derived from genomic reduction of tetraploid oat, had comparable Illumina design scores and were included in the SNP assay.

### SNP Assays

Alleles from 1,311 of the 3,072 SNP assays were clearly discriminated among the mapping progeny and/or a set of 109 diverse oat varieties, indicating a 43.7% conversion rate. Of the converted assays, 991 (44% conversion) were based on the STA, while 144 (43% conversion) and 121 (40% conversion) were derived from cDNA and DArT sequences using the CTA (Table 1). Overall, 1,169 of the 1,311 successful SNP assays (89.2%) identified polymorphic alleles in a panel of 109 diverse oat lines (Table 1, Dataset S1). Among these, approximately 20% (n = 234) of SNPs detected minor alleles (<10%). Alleles from 985 of the 1,311 assays (75.1%) segregated in at least one of the bi-parental populations (Table 1).

### Map Construction

**Figure 2:**
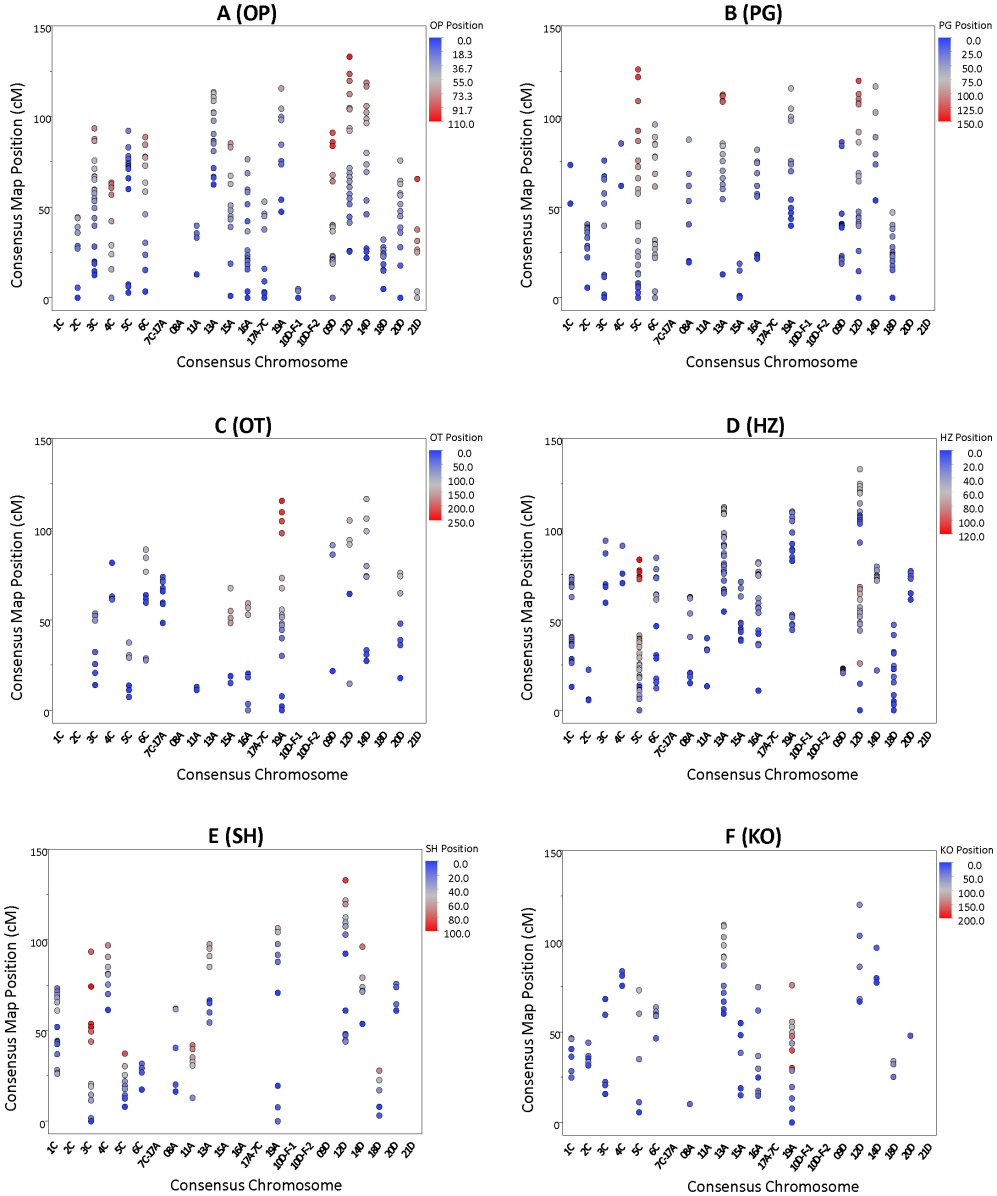
Marker density and positions for individual linkage maps (Otana/PI 260616, A; Provena/94197A1-9-2-2-2-5, B; Ogle/TAM O-301, C; Hurdal/Z-959-1, D; SolFi/HiFi, E; and Kanota/Ogle, F) in relationship to the consensus. Marker positions in the consensus map are indicated by the scales on the left axes; positions of each corresponding marker in the component maps are indicated by a color gradient described in the key.

Separate linkage maps were constructed within six RIL populations based on 320 to 647 loci (Table 2). After removing loci with unstable positions, framework maps contained 111 to 370 markers. Individual map sizes ranged from 903.8 to 1,775.7 cM, with 23 to 37 linkage groups, and marker densities ranged from 1.71 cM in SolFi/HiFi to 3.42 cM in Ogle/TAM O-301 (Fig. 2, Dataset S2). At least 100 framework loci were shared between two or more maps (Table 3), with all but 18 markers exhibiting similar order and distance. Markers with dissimilar map locations appeared to be a result of gene duplication or homology.

**Table 2:** Population size, marker statistics, map characteristics, and key traits for RIL populations used for consensus mapping.

Population	RILs	Polymorphic markers	Marker type*	Framework loci†	Map size (cM)	Largest gap (cM)	No. LG	Key traits‡
Kanota/Ogle	52	320	DS, ES	111	1,387.7	36.2	25	O/Cr/Sr/H
Hurdal/Z-595-1	51	410	BA, DS, ES	370	903.8	35.1	27	DON
Ogle/TAM O-301	49	647	AB, AF, AM, BA, BM, DS, ES, Pc, M, TLP	214	1,576.2	39.0	37	Tocol/Cr/H
Otana/PI 260616	90	487	BA, DS, ES	323	1,516.7	32.6	23	PCr
Provena/94197A1-9-2-2-2-5	97	402	BA, DS, ES	278	1,775.7	30.3	24	PCr
SolFi/HiFi	51	401	BA, DS, ES	135	1,344.9	32.9	22	BG

* AB, AF, AM, STS markers based on oat sequence; BA, genomic SNPs based on tetraploid oat; BM, genomic microsatellite based on enriched oat libraries; DS, genomic SNP based on DArT; ES, genic SNP based on EST; Pc, disease resistance phenotypic marker based on crown rust; TLP, microsatellite based on thaumatin-like pathogenesis-related protein.

† Markers identified as framework markers on the final maps using MultiPoint.

‡ O, high oil; Cr, crown rust resistance; Sr, stem rust resistance; H, historic mapping population; DON, Deoxynivalenol (toxin of Fusarium head blight); Tocol, high tocopherol; PCr, partial crown rust resistance; BG, high beta-glucan.

**Table 3:** Pairwise comparison of common SNP markers across hexaploid oat populations.

RIL population	SolFi/HiFi	Provena/94197A1-9-2-2-2-5	Otana/PI 260616	Ogle/TAM O-301	Hurdal/Z-595-1
Kanota/Ogle	100	113	133	214	111
Hurdal/Z-595-1	163	141	162	178	--
Ogle/TAM O-301	203	172	201	--	--
Otana/PI 260616	164	184	--	--	--
Provena/94197A1-9-2-2-2-5	148	--	--	--	--

**Figure 3:**
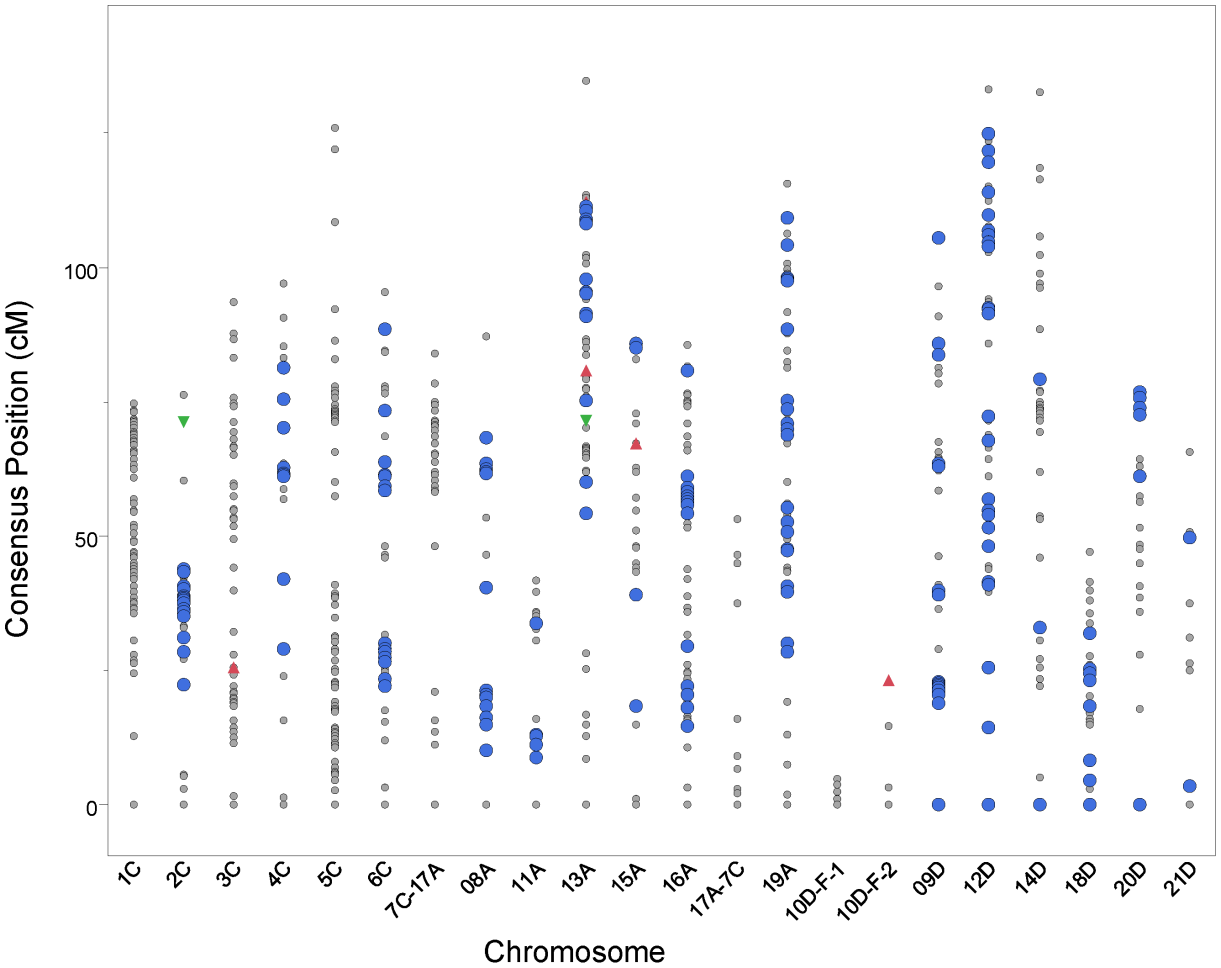
Chromosome anchoring of consensus linkage map. Grey dots indicate positions of non-anchored markers on each linkage group, while colored dots indicate positions of physically anchored SNPs (blue dots), DArTs (red up arrows), and RFLPs (green down arrows).

A consensus map was constructed from framework SNPs developed in this study and reference loci from previous maps (Table 4, Fig. 3, Fig. S1). The final consensus map contained 1,054 loci, 254 of which were not resolvable using single map solutions with limited RILs, and covered 1,838.8 cM with an average marker distance of 1.7 cM. Marker density differed between genomes, with the C genome having more markers (39.7%) than A (31.2%) or D (29.1%) (Table 4). Distribution of cDNA- and DArT-based SNPs among genomes was similar, while microsatellites mapped predominately to the A genome (64.9%). Tetraploid-derived CCDD SNPs mapped predominately to the C genome (85.8%) and resistance genes mapped exclusively to the D genome.

**Table 4:** Consensus map statistics of chromosome length, marker type, and number of markers per chromosome and genome.

Genome	Chromosome*	Length (cM)	Markers	EST SNP	DArT SNP	CCDD SNP	SSR	STS	Resistance genes	Duplicate loci
A	8A	87.1	35	28	8	0	1	0	0	2
	11A	41.9	24	20	2	0	2	1	0	0
	13A	134.6	64	50	8	0	6	0	0	1
	15A	85.7	31	26	3	0	2	0	0	4
	16A	85.6	85	75	5	0	5	0	0	1
	17A-7C	53.1	11	10	1	0	0	0	0	3
	19A	115.5	78	58	13	0	7	1	0	1
*Total*		*603.5*	*328*	*267*	*40*	*0*	*23*	*2*	*0*	*12*
										
C	1C	74.8	89	80	8	0	1	0	0	0
	2C	76.2	64	53	9	0	2	0	0	0
	3C	93.5	55	45	7	2	1	1	0	0
	4C	97.0	26	19	6	1	0	0	0	0
	5C	126.0	94	85	6	1	2	0	0	3
	6C	95.4	52	41	10	0	1	0	0	1
	7C-17A	83.9	43	33	7	2	1	0	0	1
*Total*		*646.8*	*417*	*356*	*47*	*6*	*8*	*1*	*0*	*5*
D	9D	105.4	100	84	11	0	2	0	3	4
	10Da	4.8	8	8	0	0	0	0	0	0
	10Db	23.2	4	2	0	0	0	0	2	0
	12D	133.1	63	50	13	0	0	0	0	1
	14D	132.5	48	41	4	0	3	0	0	1
	18D	47.2	44	40	1	0	0	0	0	1
	20D	76.7	28	23	4	1	0	0	0	0
	21D	65.6	11	8	3	0	0	0	0	0
*Total*		*588.5*	*306*	*256*	*36*	*1*	*5*	*0*	*5*	*7*
Total		1,838.8	1,053	879	123	7	36	3	5	12

* Chromosome anchoring of 8A, 17A-7C, and 7C-17A, are based on alignments from previous work; all other chromosomes are based on monosomic hybrid deletion analysis.

Twelve SNP loci that mapped to the A genome in at least two populations mapped to the C genome (five) or D genome (seven) in the remaining populations, resulting in two consensus map positions for these markers. Notably, four loci mapped to both 15A and 9D, with conserved ordering between groups (Fig. S2). In addition, one locus on 8A mapped to 5C while a different locus on 8A mapped to 14D. These results provide evidence of homoeologous relationships between chromosomes.

### Chromosome Assignment

Forty-five F_1_ deletion hybrids were interrogated with GoldenGate SNP assays and alleles from 234 loci were used to anchor 15 of the 21 linkage groups to chromosomes. Stocks representing 2C, 6C, 9D, 12D, and 16A produced the most robust data (Fig. S3), with a mean of 26 anchors per chromosome (Fig. 2). Stocks representing 4C, 8A, 11A, 13A, 18D, and 19A produced a mean of 14.8 anchors per chromosome, and stocks for 14D, 15A, 20D, and 21D produced two, five, six and two SNP anchor loci. Heterogeneous assignment was observed for stocks representing 11A *vs.* 5C and 1C *vs.* 8A. In these cases, the linkage group was assigned to the stock with the greater number of anchored SNP markers. C-band staining of F_2_ progeny revealed substantial chromosome rearrangement, as expected in segregating aneuploid hybrids.

DArT dilution analysis revealed 200 SNP alleles having reduced hybridization signals within a monosomic stock; these were used to anchor linkage groups 3C and 10D (Fig. 2) and to confirm assignments on 9D, 10D, 3C, 13A, 14D, and 15A.

### Comparative mapping

**Figure 4:**
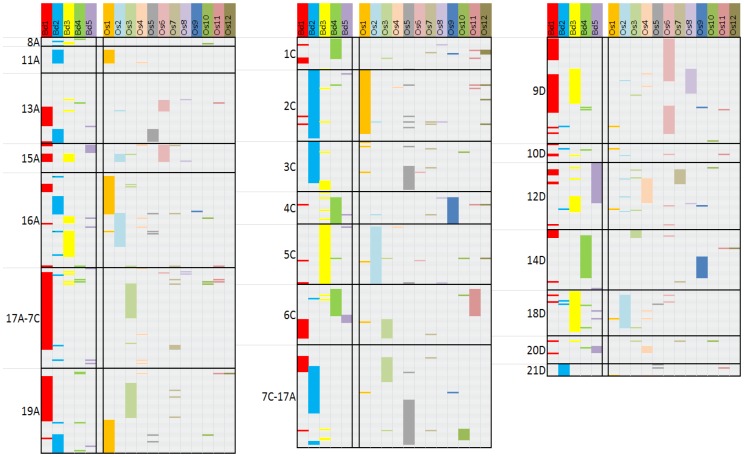
Regions of sequence similarity between SNP markers on 21 chromosomes from an oat consensus map (8A to 21D) and chromosomes from the sequenced genomes of *Brachypodium distachyon* (Bd) and *Oryza sativa* (Os). Regions of substantial colinearity have been interpolated using chromosome-specific colors.

A set of 367 SNPs showed highly significant matches (score>100 or E<5E-20) with genomes of model grass species rice and *Brachypodium*. Of these, 30 were highly repetitive and were ignored. The remaining 337 loci were colored to highlight collinearity between chromosomes (Fig. 4, Dataset S3). Although this representation is biased by the forced uniform spacing of oat markers, it demonstrates extensive regions of oat collinearity to rice and *Brachypodium* chromosomes. For example, extensive regions of oat 2C correspond with *Brachypodium* 2 and rice 1, while oat 3C matches *Brachypodium* 3 and rice 2. These and most other matches are highly consistent with known orthologies between rice and *Brachypodium* [25]. Collinearity between genomes was not perfect (Dataset S3), highlighting genetic rearrangements that are expected in evolutionarily divergent species with different ploidy levels.

## Discussion

This work describes novel methods for SNP discovery in polyploids, and a novel physical chromosome anchoring strategy. These methods were applied to develop a new high-throughput SNP platform in oat, and then to produce the first oat consensus map assigned to physical chromosomes. These resources then enabled the first integrative summary of chromosome similarities among oat, rice and *Brachypodium*.

### The first high throughput oat SNP assay

Discovery and application of SNPs in polyploid species is a relatively new challenge; therefore, we wished to compare the success of various approaches. The CTA would theoretically allow prediction of non-redundant SNPs indexed to a single reference genome. The success rate of CTA calls (29%) was lower than that of the STA-based SNPs (33%) but this is likely because some of the best CTA-based SNPs that were redundant with selected STA-based SNPs were eliminated. However, the consensus sequences from composite assemblies contained many ambiguities that confounded SNP identification, while the STA approach provided cleaner templates and was more amenable to automation. Therefore the STA was used for the majority of cDNA-based SNPs, and we recommend this strategy for future work.

The CTA was used for all DArT-based SNP calls. This choice was made because composite assemblies of DArT sequences were less complex, and because sequence redundancy among varieties was lower due to selectivity of DArT complexity reduction. It was also hoped that DArT sequences might provide a higher rate of polymorphism than cDNA-derived sequences because they contain less coding sequence. Results from the CTA suggested a slightly higher SNP rate in DArT sequences than in cDNA sequences but this was negated by a higher attrition rate after filtering. Although fewer SNPs were called in the DArT sequences, the conversion rate of called SNPs was similar to those based on cDNA.

Special attention was given to SNPs based on published DArT marker clones that could provide a bridge to DArT-based maps. Although these markers had the highest success rate, only 66 met the stringent filtering criteria. More disappointing was that only five pairs of DArT/SNP markers could be mapped in the same population; however, all corresponding pairs mapped to identical locations (Table S1) suggesting a low rate of DArT-to-SNP conversion but a high rate of success in marking identical loci. Higher conversion rates may require a strategy to access diagnostic polymorphisms at the ends of DArT sequences.

As demonstrated in previous SNP genotyping studies, homoeology and gene duplication affect hybridization and cluster resolution, complicating genotype calls and decreasing conversion from *in silico* to physical assays [19], [26], [27]. Conversion rates in this study (43.7%) were lower than in large-scale studies in barley and maize (approximately 90%) [27]–[29], likely reflecting the complex genomic structure of oat. However, variations in SNP source, population structure, and crop biology make impartial comparison of results difficult.

In this study, approximately 20% of converted SNPs detected alleles with MAF<0.10. For comparison, selected barley SNPs also showed 20% with a MAF<0.08 [18], while 16% of maize markers had MAF<0.10 across a panel of 154 diverse inbred lines [27]. These frequencies can be highly dependent on ascertainment bias. In another study, barley SNPs tested across 102 predominately European accessions revealed that 50% of SNPs were monomorphic or had MAF<0.10 [29]. The oat lines from which the current SNPs were developed represent a diverse global germplasm collection and were expected to be relatively free of ascertainment bias. More than 70% of converted oat SNPs were polymorphic in at least one of six mapping populations, while in maize, 69% of successful SNP assays were polymorphic in at least one of two mapping populations [27]. These results, compared to previous work, underline the success of *in silico* SNP identification methods used in this study.

### The first oat consensus map

Until now, it has not been possible to resolve 21 oat linkage groups or to merge individual maps into a single consensus. Previous maps in hexaploid oat have been incomplete, or have resolved to substantially more than 21 linkage groups, and the scarcity of high throughput methods providing reliable single-locus assays that segregate in multiple populations has provided only fragmentary alignment among linkage groups from different populations [11]. In the current work, SNP assays produced stable grouping and ordering in all six maps. Most encouraging was the high level of shared markers and co-linearity among maps. This result has been elusive in previous oat research due to the heterogeneity of marker assays, and because many assays have detected variants at multiple loci. The success of the current work is likely because these SNP markers have been highly filtered for consistency and reliability such that most of them directly interrogate a specific bi-allelic variant within a single defined locus.

Framework maps for all six populations were used to build the consensus map consisting of 1,054 loci defining 21 groups with a total length of 1,838 cM. This distance was similar to the previous Kanota/Ogle (1,890 cM) and Ogle/TAM O-301 (2,049 cM) maps, which were about 1,000 cM shorter than an estimated size (3,100 cM) [30]. However, previous maps likely had incorrect marker orders due to genotyping errors, inflating this estimated size [31], [32]. Based on this study, which utilized an iterative mapping approach to remove problematic loci and multiple crossovers, we estimate the total genetic length of the oat genome to be closer to 2,000 cM.

The two physically smallest chromosomes, 11A and 18D, produced short genetic maps as expected [7]. The lack of markers and short genetic distance on 17A–7C may be a result of the reciprocal translocation [33] which caused inconsistency among populations and elimination of many markers. There was no obvious explanation for lack of markers and short map distances on 10D and 21D.

### Assignment to physical oat chromosomes

Integration of genetic and physical map data in other crop species has depended on the availability of cytogenetic stocks. In hexaploid wheat, homoeologous chromosome buffering has allowed development of telocentric chromosomes, sub-arm deletion stocks, and monosomics, all of which have facilitated physical mapping [20], [21]. Although oat is also a hexaploid, chromosome rearrangement and fragmented homoeologies have resulted in weaker genomic buffering, which could explain the relative difficulties of developing and maintaining oat aneuploid stocks. In this study, monosomic hybrid deletion stocks representing 18 of the 21 oat chromosomes were developed. Although a complete monosomic series had been reported [23], monosomic progenies were not recovered for 3C, a possible indication of minimal buffering and intolerance of monosomy for this chromosome. Monosomics were also unavailable for 7C, which was nullisomic, and for 10D, for which the monosomic appeared to have shifted to 12D. C-banding analysis of monosomic 8A indicated a partial deletion of 1A, explaining the similar clustering between hybrids of these chromosome stocks. Stocks monosomic for 13A, 14D, and 17A were available in a ‘Kanota’, rather than ‘Sun-II’, background. SNP data for hybrid stocks representing these chromosomes were less informative, possibly because of a lower polymorphism rate between Ogle and TAM O-301 with Kanota, compared to Sun-II. Additionally, 17A contains a major translocation with 7C that is present in the majority of *sativa*-type oats, but lacking in *byzantina* types, including Kanota [33]. Heterozygosity for this translocation in hybrids with Kanota adversely affected aneuploid development and mapping in both chromosomes. Thus, 15 monosomic hybrids provided data used to anchor chromosomes.

Chromosome assignments of the consensus map based on chromosome deletion SNP anchoring, and to a lesser extent, the DArT dilution approach, were robust and concurred with previous reports. Fox et al. [24] used eight F_2_ plants to simulate an F_1_ hybrid and anchored 22 RFLP linkage groups to 16 chromosomes, with no associations for 3C, 4C, 6C, 10D, or 20D. Assignments in the RFLP study were made with a mean of 3.7 markers per chromosome, reflecting the scarcity of available markers at the time, as well as limitations of using segregating F_2_ progenies to simulate the critical F_1_ hybrid. In this study, clear data were not obtained for chromosomes 1C, 5C, 7C-17A, and 17A–7C, although linkage group characteristics and available marker data suggest that the Fox assignments are correct. Remaining assignments from Fox were verified by our methods. In addition, chromosomes not previously reported have now been anchored: 4C, 6C, and 20D with SNP deletion analysis and 3C and 10D with DArT dilution analysis.

Chromosome-linkage group associations highlight characteristic tendencies within each genome. Genetic conservation appeared to be strongest in the D genome, indicated by a lower level of polymorphism and the prevalence of major oat disease resistance genes (Table 4). Prevalence of disease resistance genes in one subgenome has been found in other allopolyploid species such as wheat, where the number of identified disease resistance genes was two-fold higher in the B subgenome than in A or D, fitting the “genome asymmetry” concept [34]. Evidence for a lower rate of polymorphism in the oat D genome is also provided by the putatively-homoeologous satellite chromosomes 19A and 20D [35], which have genetic lengths of 115.5 and 76.7 cM despite nearly identical physical lengths. Similarity between the A and D genomes may have biased historical marker selection in favor of the A genome, which appears to have more frequent polymorphism; this would explain the preponderance of microsatellite markers mapping to the A genome. Markers derived from *A. magna*, hypothesized to carry the C and D genomes, mapped primarily to C-genome chromosomes, again reflecting polymorphism differences between genomes. One *A. magna* SNP mapped to 20D.

Twelve SNP loci on the consensus map were assigned to more than one chromosome. In most cases, markers mapped to different genomes, exemplified by the four markers mapping to 9D and 15A. Exceptions to this were markers that mapped to chromosome 5C and two different D genome chromosomes.

### Comparative mapping in oat

Over the next few years we expect that the new SNP platform will be utilized extensively for structural and functional genomics studies. However, there will be immediate interest in identifying chromosomal positions of genes and QTLs that have been located using other maps. This was a primary reason for including Kanota/Ogle (KO) progeny, the most widely-referenced mapping population. Alignment between the recently expanded KO map [11] and the anchored consensus map was possible based on 266 SNP loci representing all 21 chromosomes (Table S1). Co-linearity between maps was strong, allowing consolidation of nine KO linkage groups in the previous map. Additionally, co-linearity between maps allowed chromosome assignments in this study to build on chromosome assignments from previous work [24]. These comparisons inevitably revealed some ongoing puzzles. For example, it was previously believed that linkage groups KO_22_44_18 and KO_24_26_34 were homoeologous, but the current map assigns these to different chromosomes within the same sub-genome (19A and 16A) while the comparison to rice and *Brachypodium* demonstrates that there are fragmented but common orthologous origins between these two chromosomes (Fig. 4). It now seems likely that substantial parts of two sub-genomic chromosomes can contain translocated homoeologous components.

### Comparison to model genomes

Identification of substantial regions of macro-colinearity between oat, rice, and *Brachypodium* provides encouragement for the use of comparative genomics to understand and utilize genome resources in oat and other related species. Although the oat genome is complex, its ancestral origins are likely intermediate between those of rice and *Brachypodium* [25]. Thus, further work on this species will assist with the understanding of evolution among grass species. Previous work that compared a DArT-based linkage map from Kanota/Ogle to the genome of *Brachypodium* [36] showed many similarities that are consistent with the current comparison (Fig. 4). For example, group KO15, equivalent to oat 2C (Table S1), matched with *Brachypodium* 2 as it does in the current work. However, there are also inconsistencies (e.g. KO 5_30 did not match well with *Brachypodium* 3 as does its equivalent oat 5C in the current work). This is likely because former comparisons were based on genomic markers with fewer and weaker orthologies than the 337 highly-informative gene-based anchors in the current work. Comparisons made in this study demonstrate orthology and colinearity on a large scale; however, there are likely to be substantial differences on the micro-synteny scale.

### Conclusion

SNP development via the discovery pipelines presented here has enabled generation of the first consensus map for the complex hexaploid oat genome, and has provided a new integrative analysis of macro-colinearity among oat, rice and *Brachypodium*. In addition, the chromosome deletion hybrid SNP anchoring strategy has enabled the first comprehensive anchoring of a genetic map to specific chromosomes. These results are a key resource for gene-based plant improvement approaches such as marker-assisted breeding, and for molecular genetics studies such as candidate gene identification and map-based cloning. This work will enable detailed exploration of genomic similarities among grasses and will contribute to research advances in other orphan crops with complex genomes.

## Materials and Methods

### DNA Libraries and Sequencing

Tissues of roots, shoots, pistillate structures, and mature embryos from 20 genotypes (Dataset S1) were used for cDNA library construction and sequencing [15]. Genomic complexity reduction was performed using *Pst*I/*Taq*I protocols on the same 20 genotypes plus an additional five lines [11]. DArT preamplification was performed using DArT-*Pst*I PCR primers and resulting amplicons were labeled with standard Roche Multiplex Identifier Tags (MID) and sequenced on the 454 GS-FLX system (Roche, Indianapolis, IN, USA).

### Chromosome-Deficient Hybrid Development

Monosomics for 13A, 14D, and 17A were derived from Kanota [37]; monosomics for remaining chromosomes were derived from Sun II [23], [38]. Monosomics for 3C and 10D were available only for DArT analysis. Monosomic hybrids were generated by crossing monosomic stocks (maternal parent) to Ogle and TAM O-301. Cytogenetic confirmation included C-band analysis of mitotic root tip cells [7], [8] from parental stocks, and presence of at least 15% micronuclei in microsporocyte counts performed on parental plants prior to hybridization and on F_1_ progenies prior to tissue collection and genotypic analysis.

### Modified *In Silico* SNP Discovery Methods

Candidate SNPs were called using three approaches (single-template (STA), composite-template (CTA), and Sanger-template) using cDNA and genomic sequences. The STA (Fig. 1) was based on Ogle, Assiniboia, TAM O-301, and Hurdal reference assemblies [15]. Templates were assembled within each variety using MIRA 3.2.0, and gsMapper (Roche) was used to map original reads onto reference assemblies. Shallow reads (depth<5) and sequences with complex or non-uniform polymorphism were eliminated. In the CTA (Fig. 1), raw reads were assembled (98% similarity) into a consensus, trimmed to regions with a depth >2 reads, and assembled into a composite template (90% similarity). Resulting consensus reads were used as a template to reassemble condensed 454 reads. Resulting assemblies, in Phrap format, were processed using the CTA, which calls all potential SNPs and tabulates depth of coverage, allele frequency, and varietal purity. Tabulated SNPs were filtered to retain only those that showed purity within varieties and diversity among varieties. The Sanger-template strategy was similar to the CTA but used non-redundant Sanger sequences from the published oat DArT marker assay [11]. Additional candidate SNPs validated earlier using high-resolution melt analysis [15] on a tetraploid genotype panel were added from the complexity-reduced genome of *A. magna* germplasm lines Ba 13-13 and #169. Sequences containing SNPs were submitted for Illumina (San Diego, CA) GoldenGate assay design, and design scores were incorporated into final SNP filtering.

### SNP Assay Properties

SNP allele interrogation was performed using two 1,536-SNP oligo pooled assays on an Illumina BeadStation using a 32-beadchip platform (Dataset S4). Allele calls were performed using GenomeStudio v.3 software and edited manually. To facilitate heterozygote calls, six controls, developed by mixing DNA from two mapping parents, were included in the assay. Homozygous clusters were evaluated based on mapping populations, which comprised few heterozygous genotypes. Resulting cluster solutions were applied to the germplasm panel and monosomic hybrid chromosome stocks, and heterozygote controls were used to refine heterozygous clusters.

### Consensus Map Construction

Individual linkage maps were constructed for subsets of six mapping populations (Table 2) using MultiPoint software (http://www.multiqtl.com) [31], [32]. Markers with highly-distorted segregation ratios (≤0.25 or ≥0.75) and ≥10% missing genotypes were removed. Preliminary grouping and ordering was conducted with *rf* threshold = 0.15. Iterative resampling was performed to remove unstable markers, and completed groups were merged end-to-end by incrementally increasing *rf*, with a final *rf* of 0.25. Iterative resampling was repeated to verify marker order and stability.

A multilocus consensus map was created using the MultiPoint ‘Full Frame’ algorithm, which employs a synchronized traveling salesperson problem approach (http://www.multiqtl.com). Consensus mapping was performed on a combined raw dataset of six individual map solutions, with data weighted based on sample size. Local analysis was used to resolve pair-wise conflicts, and a global analysis was used to finalize the map solution. The overall consensus solution was compared to an integrated map solution generated using JoinMap v. 4 software [39]. The graph builder and linkage map viewer in JMP Genomics 5.1 software (SAS Institute, Cary NC) were used to visualize solutions.

### Chromosome-Deficient SNP Anchoring

Chromosome assignment was based on previous deletion analysis methods [24], [40] but used sequence-based markers to directly interrogate F_1_ chromosome-deficient hybrids. SNP loci with polymorphism between a paternal parent (Ogle or TAM O-301) and a maternal parent background (Sun II or Kanota) were analyzed across chromosome-deficient hybrids. Alleles present on a critical chromosome and having a hemizygous genotype (clustering with a parental allele) were used to anchor linkage groups to chromosomes (Fig. S3).

SNP deletion results were confirmed by DArT dilution analysis [11]. Hybridization intensity of each line to each DArT representation based on background (Sun II or Kanota) was determined using DArTsoft v. 7.3 software. Representations with variable hybridization intensity within a background were analyzed using fuzzy k-means to cluster hybridization intensities. Signals between distribution tails were considered dose-affected representations compared to the complete dose normally present. The resulting data matrix was used to anchor DArT markers from the Ogle/TAM O-301 and Kanota/Ogle linkage maps to chromosomes. DArT/SNP linkages between maps were then used to align anchored SNP loci with DArT markers. The graph builder in JMP Genomics 5.1 was used to visualize chromosome anchors along the consensus map.

### Comparative mapping

Design sequences from all mapped SNPs were matched against pseudomolecule representations of complete genomes from rice (*Oryza sativa* L., release 6.1; http://rice.plantbiology.msu.edu) and *Brachypodium distachyon* L. (release 2.1; http://www.brachypodium.org) using nucleotide (BLASTn) and translated (tBLASTx) instances of NCBI BLAST v 2.2.24 [41]. Matches were filtered to retain those with a bit score >100 and/or an expectation <5E-20, and to keep only the best match for each integer-rounded midpoint (Mb) per chromosome. SNPs matching more than three *Brachypodium* chromosomes were ignored, and remaining matches were used to infer regions of oat chromosomes having sequential orthology to those of the model genomes.

## Supporting Information

Figure S1 **A 21-chromosome anchored consensus map of oat.**
(TIF)

Figure S2 **Colinearity of SNP loci mapping to chromosomes 9D and 15A.**
(TIF)

Figure S3 **SNP deletion analysis of monosomic hybrid stocks representing chromosomes 6C and 9D.**
(TIF)

Table S1 Correspondence of consensus chromosomes with published KO linkage groups.
(DOCX)

Dataset S1 **Pedigree data for 20 genotypes selected for transcriptome sequencing and SNP identification and for 109 genotypes selected to represent genetic diversity in North American oat germplasm.**
(XLSX)

Dataset S2 **Consensus map and component maps from six bi-parental mapping populations, including original SNP genotype calls for component mapping populations.**
(XLSX)

Dataset S3 **Details for regions of sequence similarity between SNP markers on 21 chromosomes from an oat consensus map and chromosomes from the sequenced genomes of** ***Brachypodium distachyon*** **and** ***Oryza sativa*** **.**
(XLSX)

Dataset S4 **Sequences for** ***in silico*** **SNP assays analyzed with the GoldenGate genotyping platform.**
(XLSX)
