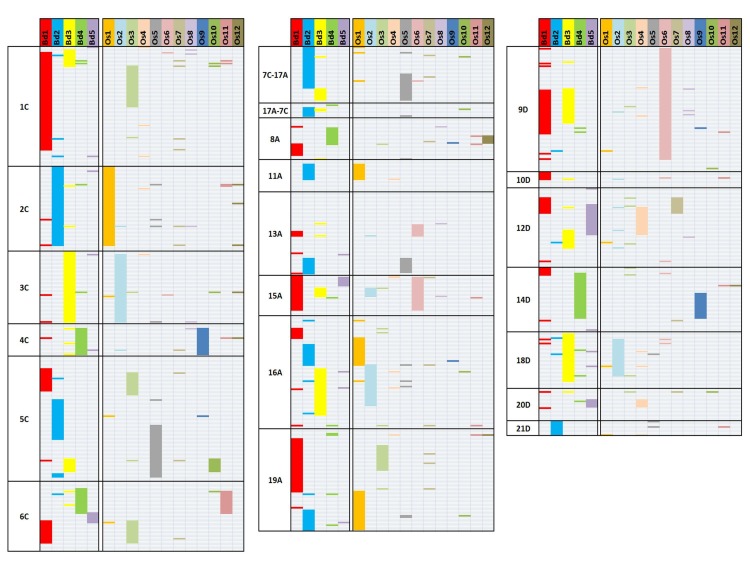# Correction: SNP Discovery and Chromosome Anchoring Provide the First Physically-Anchored Hexaploid Oat Map and Reveal Synteny with Model Species

**DOI:** 10.1371/annotation/9b2ca31c-0aca-44b1-84a1-8bdf8ded7439

**Published:** 2013-10-11

**Authors:** Rebekah E. Oliver, Nicholas A. Tinker, Gerard R. Lazo, Shiaoman Chao, Eric N. Jellen, Martin L. Carson, Howard W. Rines, Donald E. Obert, Joseph D. Lutz, Irene Shackelford, Abraham B. Korol, Charlene P. Wight, Kyle M. Gardner, Jiro Hattori, Aaron D. Beattie, Åsmund Bjørnstad, J. Michael Bonman, Jean-Luc Jannink, Mark E. Sorrells, Gina L. Brown-Guedira, Jennifer W. Mitchell Fetch, Stephen A. Harrison, Catherine J. Howarth, Amir Ibrahim, Frederic L. Kolb, Michael S. McMullen, J. Paul Murphy, Herbert W. Ohm, Brian G. Rossnagel, Weikai Yan, Kelci J. Miclaus, Jordan Hiller, Peter J. Maughan, Rachel R. Redman Hulse, Joseph M. Anderson, Emir Islamovic, Eric W. Jackson

The published Figure 4 is incorrect. Please view the corrected Figure 4 here: 

**Figure:**