# Supplementary figures and images for: SNP Discovery and Chromosome Anchoring Provide the First Physically-Anchored Hexaploid Oat Map and Reveal Synteny with Model Species
Source: PLoS One. 2013 Mar 22;8(3):e58068. doi: 10.1371/journal.pone.0058068 (PMC3606164; doi:10.1371/journal.pone.0058068)

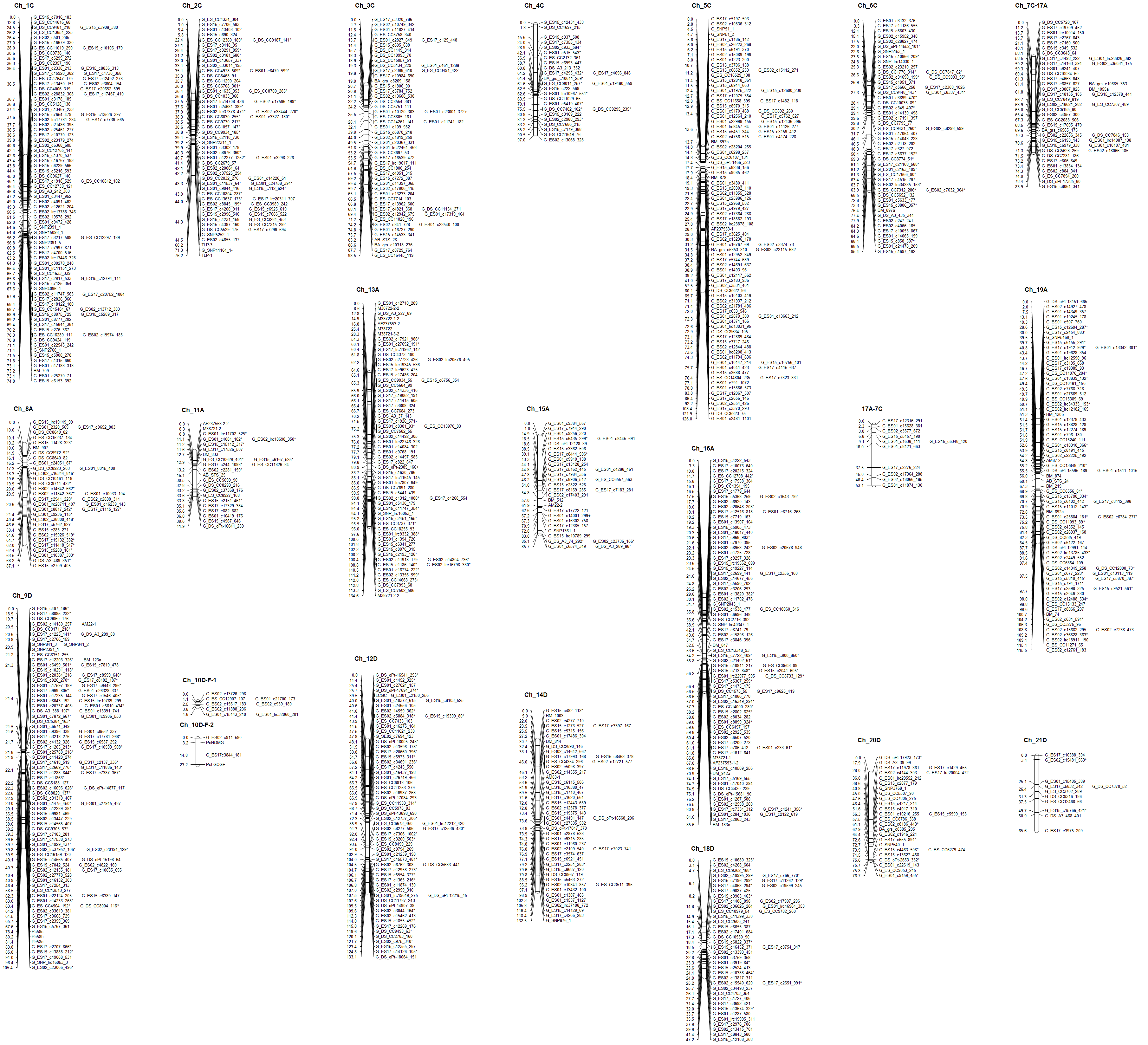

Supplement: Figure S1 — A 21-chromosome anchored consensus map of oat. (TIF) [file pone.0058068.s001.tif]

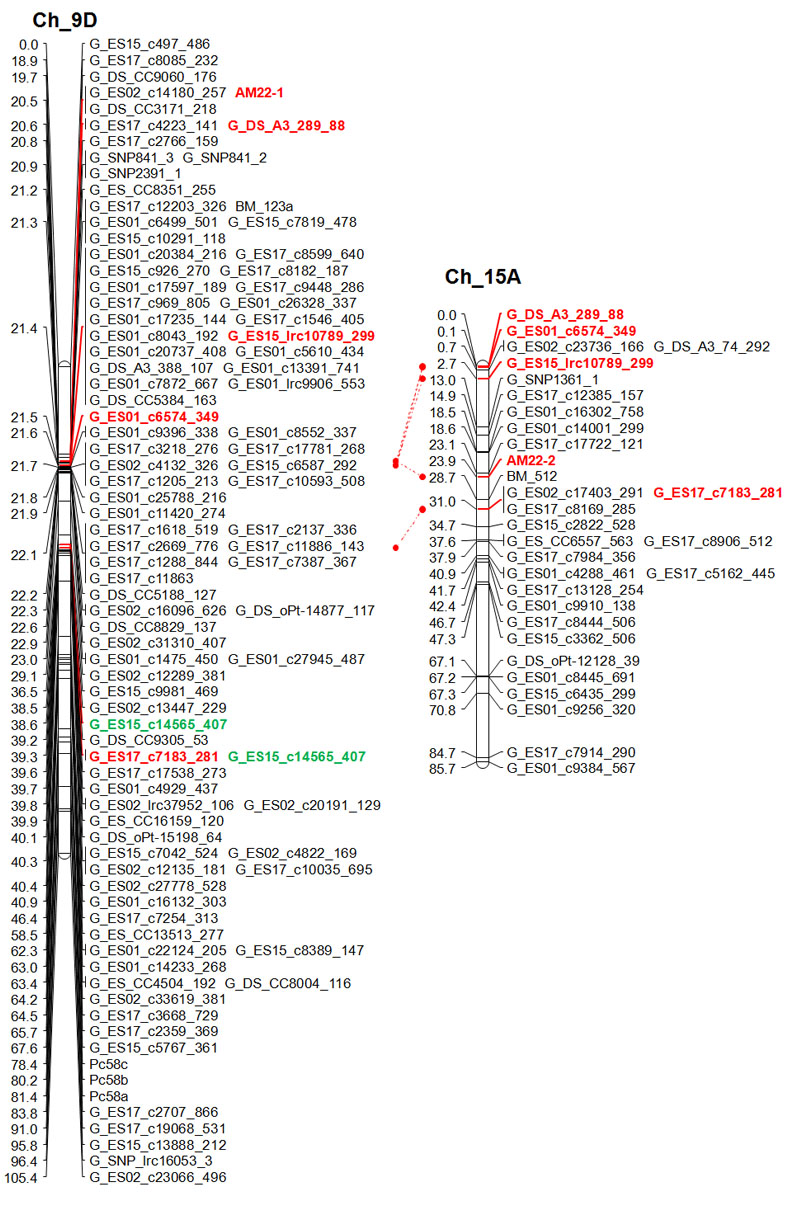

Supplement: Figure S2 — Colinearity of SNP loci mapping to chromosomes 9D and 15A. (TIF) [file pone.0058068.s002.tif]

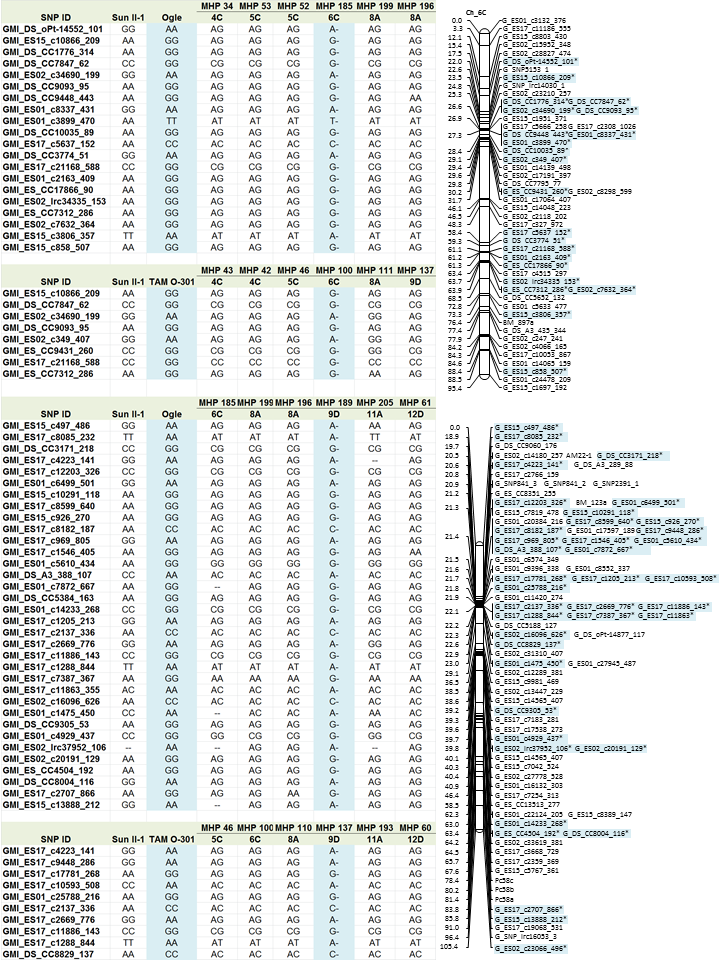

Supplement: Figure S3 — SNP deletion analysis of monosomic hybrid stocks representing chromosomes 6C and 9D. (TIF) [file pone.0058068.s003.tif]
