# Supplementary material for: SNP Discovery and Chromosome Anchoring Provide the First Physically-Anchored Hexaploid Oat Map and Reveal Synteny with Model Species
Source: PLoS One. 2013 Mar 22;8(3):e58068. doi: 10.1371/journal.pone.0058068 (PMC3606164; doi:10.1371/journal.pone.0058068)
Supplement: Table S1 — Correspondence of consensus chromosomes with published KO linkage groups. (DOCX) [file pone.0058068.s004.docx]

| Table S2. Correspondence of oat consensus map to the published Kanota/Ogle linkage map (15) | | | | |
| --- | --- | --- | --- | --- |
| **Locus** | **Chromosome** | **Position** | **KO Linkage Map** | **KO Position** |
| GMI_ES15_c3908_380 | Ch_1C | 24.529 | KO_21_46_31_40 | 29.9 |
| GMI_ES15_c8836_313 | Ch_1C | 36.403 | KO_21_46_31_40 | 27.5 |
| GMI_ES01_c13467_233 | Ch_1C | 37.442 | KO_21_46_31_40 | 27.5 |
| GMI_ES15_c7654_479 | Ch_1C | 37.568 | KO_21_46_31_40 | 27.5 |
| GMI_ES17_c1918_529 | Ch_1C | 46.097 | KO_21_46_31_40 | 71.8 |
| GMI_DS_A3_242_303 | Ch_1C | 46.781 | KO_21_46_31_40 | 71.8 |
| GMI_ES02_c12621_204 | Ch_1C | 49.281 | KO_11_41_20_45 | 6.03 |
| GMI_ES01_c9472_428 | Ch_1C | 51.981 | KO_11_41_20_45 | 8.27 |
| GMI_ES17_c4700_516 | Ch_1C | 62.531 | KO_37 | 3.52 |
| GMI_ES01_lrc11151_273 | Ch_1C | 65.448 | KO_21_46_31_40 | 98.4 |
| GMI_ES02_c19974_185 | Ch_1C | 70.267 | KO_11_41_20_45 | 6.03 |
| GMI_ES17_c1315_660 | Ch_1C | 71.823 | KO_11_41_20_45 | 8.27 |
| GMI_ES01_c17183_318 | Ch_1C | 73.057 | KO_11_41_20_45 | 13.7 |
| GMI_ES01_c13403_102 | Ch_2C | 5.373 | KO_24_26_34 | 2.32 |
| GMI_ES17_c3418_95 | Ch_2C | 27.143 | KO_15 | 80.8 |
| GMI_ES01_c13657_337 | Ch_2C | 32.982 | KO_15 | 81.3 |
| GMI_ES17_lrc14708_436 | Ch_2C | 37.454 | KO_15 | 48.8 |
| GMI_ES01_c24681_389 | Ch_2C | 37.637 | KO_15 | 49.1 |
| GMI_ES02_c8676_360 | Ch_2C | 40.278 | KO_15 | 50.9 |
| GMI_ES01_c12277_1252 | Ch_2C | 40.658 | KO_15 | 49 |
| GMI_ES01_c3298_226 | Ch_2C | 40.658 | KO_15 | 48.9 |
| GMI_ES02_c20004_64 | Ch_2C | 42.139 | KO_15 | 59.7 |
| GMI_ES02_c37525_294 | Ch_2C | 42.171 | KO_15 | 59.7 |
| GMI_ES01_c14226_61 | Ch_2C | 43.319 | KO_15 | 57.6 |
| GMI_ES01_c9044_416 | Ch_2C | 43.319 | KO_15 | 57.6 |
| GMI_ES01_c11537_64 | Ch_2C | 43.319 | KO_15 | 57.6 |
| GMI_ES17_lrc20311_707 | Ch_2C | 43.99 | KO_15 | 56.9 |
| GMI_ES02_c8845_199 | Ch_2C | 43.99 | KO_15 | 57.6 |
| GMI_ES02_c4655_137 | Ch_2C | 44.486 | KO_15 | 56.9 |
| GMI_ES01_c11827_414 | Ch_3C | 11.484 | KO_36 | 9.36 |
| GMI_ES17_c125_448 | Ch_3C | 13.728 | KO_36 | 23.9 |
| GMI_ES15_c605_638 | Ch_3C | 14.427 | KO_36 | 23.4 |
| GMI_ES17_c5784_752 | Ch_3C | 20.944 | KO_36 | 15 |
| GMI_ES01_c109_982 | Ch_3C | 32.133 | KO_36 | 18.8 |
| GMI_ES01_c14397_365 | Ch_3C | 59.361 | KO_42 | 8.12 |
| GMI_ES02_c17906_415 | Ch_3C | 59.715 | KO_42 | 9.11 |
| GMI_ES01_c17319_464 | Ch_3C | 69.389 | KO_42 | 0 |
| GMI_ES01_c22540_100 | Ch_3C | 74.175 | KO_42 | 0.57 |
| GMI_ES15_c337_508 | Ch_4C | 15.647 | KO_32 | 15 |
| GMI_ES17_c7355_434 | Ch_4C | 24.004 | KO_32 | 19.7 |
| GMI_ES01_c515_543 | Ch_4C | 42.096 | KO_32 | 23.6 |
| GMI_ES02_c13068_328 | Ch_4C | 97.023 | KO_19+25+27 | 35.7 |
| GMI_ES17_c5197_503 | Ch_5C | 0 | KO_46 | 3.41 |
| GMI_ES02_c10836_312 | Ch_5C | 2.843 | KO_5_30 | 68.2 |
| GMI_ES02_c26223_268 | Ch_5C | 6.028 | KO_5_30 | 68.4 |
| GMI_ES02_c15089_196 | Ch_5C | 7.112 | KO_5_30 | 68.8 |
| GMI_ES15_c12818_361 | Ch_5C | 11.352 | KO_5_30 | 58.2 |
| GMI_ES15_c6914_663 | Ch_5C | 11.521 | KO_5_30 | 55.3 |
| GMI_ES15_c12600_230 | Ch_5C | 12.42 | KO_5_30 | 53.5 |
| GMI_ES01_c11975_322 | Ch_5C | 12.42 | KO_5_30 | 49.4 |
| GMI_ES15_c8970_315 | Ch_5C | 12.888 | KO_6 | 87.3 |
| GMI_ES01_c12564_210 | Ch_5C | 13.435 | KO_5_30 | 50.9 |
| GMI_ES01_c11126_277 | Ch_5C | 13.554 | KO_5_30 | 53.2 |
| GMI_ES01_c22998_155 | Ch_5C | 13.554 | KO_5_30 | 49.3 |
| GMI_ES15_c12436_395 | Ch_5C | 13.554 | KO_5_30 | 53.5 |
| GMI_ES01_lrc8457_64 | Ch_5C | 13.554 | KO_5_30 | 49.3 |
| GMI_ES15_c5451_344 | Ch_5C | 13.554 | KO_5_30 | 49.3 |
| GMI_ES02_c4756_515 | Ch_5C | 13.636 | KO_5_30 | 50.9 |
| GMI_ES02_c28204_255 | Ch_5C | 14.029 | KO_5_30 | 49.3 |
| GMI_ES01_c6298_257 | Ch_5C | 14.094 | KO_5_30 | 53.4 |
| GMI_DS_oPt-1466_323 | Ch_5C | 17.362 | KO_5_30 | 50.9 |
| GMI_ES15_c8238_156 | Ch_5C | 17.672 | KO_5_30 | 50.9 |
| GMI_ES02_c14691_637 | Ch_5C | 38.445 | KO_5_30 | 27.9 |
| GMI_ES01_c12117_562 | Ch_5C | 39.241 | KO_5_30 | 27.8 |
| GMI_ES17_c2183_936 | Ch_5C | 41.028 | KO_5_30 | 22.6 |
| GMI_ES02_c31937_212 | Ch_5C | 71.086 | KO_5_30 | 5.9 |
| GMI_ES02_c21781_486 | Ch_5C | 71.371 | KO_5_30 | 5.9 |
| GMI_ES01_c2879_300 | Ch_5C | 72.263 | KO_5_30 | 5.98 |
| GMI_ES01_c13663_212 | Ch_5C | 72.307 | KO_5_30 | 5.24 |
| GMI_ES01_lrc13031_95 | Ch_5C | 72.573 | KO_5_30 | 5.24 |
| GMI_ES17_c12869_484 | Ch_5C | 73.064 | KO_5_30 | 1.18 |
| GMI_ES01_lrc8208_413 | Ch_5C | 73.621 | KO_5_30 | 1.35 |
| GMI_ES15_c10756_401 | Ch_5C | 75.699 | KO_5_30 | 1.18 |
| GMI_ES01_c10147_214 | Ch_5C | 75.699 | KO_5_30 | 1.18 |
| GMI_ES15_c3688_477 | Ch_5C | 75.739 | KO_5_30 | 1.33 |
| GMI_ES17_c2656_146 | Ch_5C | 86.354 | KO_5_30 | 1.42 |
| GMI_ES02_c2554_426 | Ch_5C | 92.19 | KO_5_30 | 1.66 |
| GMI_ES15_c8803_430 | Ch_6C | 12.068 | KO_29_43 | 43.5 |
| GMI_DS_oPt-14552_101 | Ch_6C | 22.018 | KO_29_43 | 42.3 |
| GMI_ES15_c10866_209 | Ch_6C | 23.495 | KO_29_43 | 42 |
| GMI_ES02_c2118_202 | Ch_6C | 46.45 | KO_7_10_28 | 117 |
| GMI_ES01_c2163_409 | Ch_6C | 61.218 | KO_7_10_28 | 116 |
| GMI_DS_A3_435_344 | Ch_6C | 77.434 | KO_7_10_28 | 145 |
| GMI_ES01_c14065_159 | Ch_6C | 84.607 | KO_7_10_28 | 155 |
| GMI_ES01_c24478_209 | Ch_6C | 88.465 | KO_7_10_28 | 156 |
| GMI_ES17_c12316_291 | Ch_17A-7C | 0 | KO_1_3_38_X2 | 15.1 |
| GMI_ES01_c15628_381 | Ch_17A-7C | 2.287 | KO_1_3_38_X2 | 15.3 |
| GMI_ES01_c8121_663 | Ch_17A-7C | 16.024 | KO_1_3_38_X2 | 12.1 |
| GMI_ES15_lrc19149_99 | Ch_8A | 0 | KO_16_23 | 80.1 |
| GMI_ES17_c9652_803 | Ch_8A | 10 | KO_16_23 | 69.1 |
| GMI_ES15_c285_271 | Ch_8A | 53.392 | KO_16_23 | 86.7 |
| GMI_ES01_lrc11702_525 | Ch_11A | 8.765 | KO_4_12_13 | 60.8 |
| GMI_ES17_c17329_384 | Ch_11A | 35.073 | KO_4_12_13 | 37.9 |
| GMI_ES17_c882_882 | Ch_11A | 35.568 | KO_4_12_13 | 37.5 |
| GMI_ES01_c10419_176 | Ch_11A | 35.961 | KO_4_12_13 | 37.5 |
| GMI_DS_oPt-16041_239 | Ch_11A | 41.867 | KO_4_12_13 | 32.6 |
| GMI_DS_A3_227_89 | Ch_13A | 12.823 | KO_4_12_13 | 129 |
| GMI_ES02_c17921_986 | Ch_13A | 54.327 | KO_6 | 15.1 |
| GMI_ES01_c27692_191 | Ch_13A | 60.051 | KO_6 | 15.3 |
| GMI_ES02_c27723_426 | Ch_13A | 62.227 | KO_6 | 30 |
| GMI_ES15_lrc19345_536 | Ch_13A | 62.248 | KO_6 | 31.3 |
| GMI_ES17_lrc9623_475 | Ch_13A | 64.555 | KO_6 | 32.4 |
| GMI_ES02_c14336_416 | Ch_13A | 65.905 | KO_6 | 32.4 |
| GMI_DS_A3_37_143 | Ch_13A | 70.245 | KO_6 | 35.1 |
| GMI_ES17_c1926_571 | Ch_13A | 71.463 | KO_6 | 44.8 |
| GMI_ES01_c8301_93 | Ch_13A | 75.178 | KO_6 | 47.4 |
| GMI_ES02_c14492_305 | Ch_13A | 75.338 | KO_6 | 58.2 |
| GMI_DS_oPt-2385_166 | Ch_13A | 80.867 | KO_6 | 65.9 |
| GMI_ES15_c8970_315 | Ch_13A | 102.269 | KO_6 | 87.3 |
| GMI_ES15_c2193_426 | Ch_13A | 108.216 | KO_6 | 96.1 |
| GMI_ES02_c11918_179 | Ch_13A | 108.364 | KO_6 | 95.2 |
| GMI_ES02_c14804_736 | Ch_13A | 108.397 | KO_6 | 96.1 |
| GMI_ES02_lrc16798_330 | Ch_13A | 108.771 | KO_6 | 96 |
| GMI_ES01_c16774_222 | Ch_13A | 110.49 | KO_6 | 95.2 |
| GMI_ES02_c13356_599 | Ch_13A | 111.179 | KO_6 | 96.1 |
| GMI_ES01_c9256_320 | Ch_15A | 14.913 | KO_7_10_28 | 14 |
| GMI_DS_oPt-12128_39 | Ch_15A | 18.612 | KO_7_10_28 | 18.3 |
| GMI_ES02_c17403_291 | Ch_15A | 54.765 | KO_7_10_28 | 70.5 |
| GMI_ES17_c8169_285 | Ch_15A | 54.765 | KO_7_10_28 | 70.9 |
| GMI_ES01_c14001_299 | Ch_15A | 67.168 | KO_7_10_28 | 70.5 |
| GMI_ES01_c6574_349 | Ch_15A | 85.673 | KO_17 | 30.1 |
| GMI_DS_A3_289_88 | Ch_15A | 85.747 | KO_17 | 30.4 |
| GMI_ES17_c17558_304 | Ch_16A | 15.8 | KO_24_26_34 | 45.4 |
| GMI_ES17_c1779_644 | Ch_16A | 16.487 | KO_24_26_34 | 39.5 |
| GMI_ES15_c5368_259 | Ch_16A | 17.542 | KO_24_26_34 | 39 |
| GMI_ES02_c6920_143 | Ch_16A | 17.658 | KO_24_26_34 | 35.3 |
| GMI_ES15_c7719_163 | Ch_16A | 18.182 | KO_24_26_34 | 39.2 |
| GMI_ES01_c13907_104 | Ch_16A | 19.162 | KO_24_26_34 | 37.5 |
| GMI_ES01_c18017_440 | Ch_16A | 20.253 | KO_24_26_34 | 35.3 |
| GMI_ES01_c7970_395 | Ch_16A | 21.596 | KO_24_26_34 | 32.8 |
| GMI_ES01_c1725_728 | Ch_16A | 23.222 | KO_24_26_34 | 32.4 |
| GMI_ES15_c19227_114 | Ch_16A | 24.502 | KO_24_26_34 | 31.8 |
| GMI_ES17_c2699_441 | Ch_16A | 24.618 | KO_24_26_34 | 31.8 |
| GMI_ES02_c14677_456 | Ch_16A | 24.638 | KO_24_26_34 | 32.3 |
| GMI_ES17_c5590_702 | Ch_16A | 24.826 | KO_24_26_34 | 31.8 |
| GMI_ES02_c3206_293 | Ch_16A | 26.198 | KO_24_26_34 | 20.9 |
| GMI_ES02_c11702_476 | Ch_16A | 30.071 | KO_24_26_34 | 5.47 |
| GMI_ES02_c1538_477 | Ch_16A | 35.822 | KO_24_26_34 | 4.44 |
| GMI_ES01_c6696_348 | Ch_16A | 35.847 | KO_24_26_34 | 4.72 |
| GMI_ES17_c5367_259 | Ch_16A | 56.235 | KO_11_41_20_45 | 83.5 |
| GMI_ES17_c9625_419 | Ch_16A | 56.516 | KO_11_41_20_45 | 83.5 |
| GMI_ES02_c16349_294 | Ch_16A | 56.987 | KO_11_41_20_45 | 79.6 |
| GMI_ES01_c8899_324 | Ch_16A | 59.041 | KO_11_41_20_45 | 81.4 |
| GMI_ES17_c3200_273 | Ch_16A | 61.027 | KO_11_41_20_45 | 77.9 |
| GMI_ES01_c233_61 | Ch_16A | 61.086 | KO_11_41_20_45 | 77.9 |
| GMI_ES17_c786_412 | Ch_16A | 61.086 | KO_11_41_20_45 | 76.1 |
| GMI_ES17_c1612_641 | Ch_16A | 61.753 | KO_11_41_20_45 | 75.8 |
| GMI_ES17_c5169_555 | Ch_16A | 74.14 | KO_11_41_20_45 | 55.6 |
| GMI_ES01_c17040_394 | Ch_16A | 74.662 | KO_11_41_20_45 | 56 |
| GMI_ES01_c284_1036 | Ch_16A | 81.596 | KO_11_41_20_45 | 27.2 |
| GMI_ES17_c2122_619 | Ch_16A | 81.623 | KO_11_41_20_45 | 26.7 |
| GMI_ES17_c2063_243 | Ch_16A | 81.623 | KO_11_41_20_45 | 26.7 |
| GMI_DS_oPt-13151_665 | Ch_19A | 0 | KO_22_44_18 | 165 |
| GMI_ES02_c14927_478 | Ch_19A | 1.972 | KO_22_44_18 | 165 |
| GMI_ES01_c14349_357 | Ch_19A | 7.542 | KO_22_44_18 | 156 |
| GMI_ES01_c19245_178 | Ch_19A | 13.062 | KO_22_44_18 | 156 |
| GMI_ES01_c507_760 | Ch_19A | 19.294 | KO_22_44_18 | 150 |
| GMI_ES15_c12694_287 | Ch_19A | 28.588 | KO_22_44_18 | 145 |
| GMI_ES01_c13342_301 | Ch_19A | 40.768 | KO_22_44_18 | 135 |
| GMI_ES17_c1912_929 | Ch_19A | 40.768 | KO_22_44_18 | 132 |
| GMI_ES01_lrc12590_96 | Ch_19A | 43.686 | KO_22_44_18 | 130 |
| GMI_ES02_c7768_318 | Ch_19A | 49.539 | KO_22_44_18 | 117 |
| GMI_ES01_c27869_512 | Ch_19A | 49.738 | KO_22_44_18 | 117 |
| GMI_ES01_c10310_366 | Ch_19A | 52.648 | KO_22_44_18 | 114 |
| GMI_ES15_c8191_415 | Ch_19A | 53.937 | KO_7_10_28 | 156 |
| GMI_ES02_c22225_492 | Ch_19A | 54.154 | KO_22_44_18 | 90.5 |
| GMI_DS_oPt-15595_189 | Ch_19A | 55.473 | KO_22_44_18 | 91.2 |
| GMI_ES01_c1511_1015 | Ch_19A | 55.529 | KO_22_44_18 | 90.4 |
| GMI_ES01_c25884_181 | Ch_19A | 73.48 | KO_22_44_18 | 74.8 |
| GMI_ES02_c6784_277 | Ch_19A | 73.489 | KO_22_44_18 | 78.4 |
| GMI_ES02_c4352_145 | Ch_19A | 75.826 | KO_22_44_18 | 75.5 |
| GMI_ES02_c631_591 | Ch_19A | 104.235 | KO_22_44_18 | 1.22 |
| GMI_ES02_c36828_363 | Ch_19A | 109.225 | KO_22_44_18 | 2 |
| GMI_DS_A3_289_88 | Ch_9D | 20.649 | KO_17 | 30.4 |
| GMI_ES17_c2766_159 | Ch_9D | 20.825 | KO_17 | 39.4 |
| GMI_ES17_c12203_326 | Ch_9D | 21.253 | KO_17 | 38.9 |
| GMI_ES15_c7819_478 | Ch_9D | 21.267 | KO_17 | 36.3 |
| GMI_ES15_c10291_118 | Ch_9D | 21.267 | KO_17 | 38.5 |
| GMI_ES01_c20384_216 | Ch_9D | 21.351 | KO_17 | 43.4 |
| GMI_ES15_c926_270 | Ch_9D | 21.353 | KO_17 | 38.5 |
| GMI_ES17_c8182_187 | Ch_9D | 21.353 | KO_17 | 43.3 |
| GMI_ES17_c9448_286 | Ch_9D | 21.445 | KO_17 | 38.5 |
| GMI_ES01_c8043_192 | Ch_9D | 21.446 | KO_17 | 39.4 |
| GMI_ES01_c5610_434 | Ch_9D | 21.447 | KO_17 | 43.5 |
| GMI_DS_A3_388_107 | Ch_9D | 21.447 | KO_17 | 43.3 |
| GMI_ES01_c13391_741 | Ch_9D | 21.447 | KO_17 | 43.3 |
| GMI_ES01_lrc9906_553 | Ch_9D | 21.447 | KO_17 | 43.4 |
| GMI_ES01_c20737_408 | Ch_9D | 21.447 | KO_17 | 38.5 |
| GMI_ES01_c7872_667 | Ch_9D | 21.447 | KO_17 | 44.2 |
| GMI_ES01_c9396_338 | Ch_9D | 21.589 | KO_17 | 36.3 |
| GMI_ES01_c8552_337 | Ch_9D | 21.648 | KO_17 | 38.5 |
| GMI_ES02_c4132_326 | Ch_9D | 21.702 | KO_17 | 43.3 |
| GMI_ES17_c17781_268 | Ch_9D | 21.702 | KO_17 | 43.5 |
| GMI_ES15_c6587_292 | Ch_9D | 21.702 | KO_17 | 43 |
| GMI_ES17_c3218_276 | Ch_9D | 21.702 | KO_17 | 43.5 |
| GMI_ES01_c25788_216 | Ch_9D | 21.774 | KO_17 | 43.6 |
| GMI_ES02_c16096_626 | Ch_9D | 22.34 | KO_17 | 37.2 |
| GMI_ES01_c27945_487 | Ch_9D | 23.014 | KO_17 | 36.3 |
| GMI_ES02_lrc37952_106 | Ch_9D | 39.819 | KO_17 | 36.3 |
| GMI_ES17_c10035_695 | Ch_9D | 40.319 | KO_7_10_28 | 70.5 |
| GMI_ES02_c27778_528 | Ch_9D | 40.403 | KO_17 | 38.6 |
| GMI_ES15_c8389_147 | Ch_9D | 62.32 | KO_17 | 38.8 |
| GMI_ES17_c2707_866 | Ch_9D | 83.763 | KO_17 | 3.02 |
| GMI_ES15_c13888_212 | Ch_9D | 85.763 | KO_17 | 3.01 |
| GMI_ES02_c13726_298 | Ch_10D-F-1 | 0 | KO_19+25+27 | 21.5 |
| GMI_ES02_c11888_236 | Ch_10D-F-1 | 3.772 | KO_19+25+27 | 1.24 |
| GMI_ES01_c15143_210 | Ch_10D-F-1 | 4.827 | KO_19+25+27 | 4.49 |
| GMI_ES01_c4452_325 | Ch_12D | 14.41 | KO_47 | 6.41 |
| GMI_ES01_c27024_157 | Ch_12D | 25.442 | KO_47 | 3.47 |
| GMI_ES02_c7694_423 | Ch_12D | 47.777 | KO_2 | 69.2 |
| GMI_ES02_c16987_268 | Ch_12D | 66.628 | KO_2 | 59.9 |
| GMI_DS_oPt-17084_293 | Ch_12D | 66.871 | KO_2 | 59.9 |
| GMI_DS_oPt-13898_690 | Ch_12D | 71.41 | KO_2 | 57.6 |
| GMI_ES17_c12958_273 | Ch_12D | 104.709 | KO_2 | 34.4 |
| GMI_DS_oPt-12215_45 | Ch_12D | 107.542 | KO_2 | 32.6 |
| GMI_ES02_c15462_413 | Ch_12D | 112.254 | KO_2 | 19.2 |
| GMI_ES02_c975_340 | Ch_12D | 121.681 | KO_2 | 12 |
| GMI_ES15_c482_113 | Ch_14D | 0 | KO_39 | 0.52 |
| GMI_ES17_c3397_167 | Ch_14D | 23.501 | KO_5_30 | 108 |
| GMI_ES15_c1273_527 | Ch_14D | 23.501 | KO_5_30 | 108 |
| GMI_ES15_c5315_156 | Ch_14D | 25.516 | KO_5_30 | 108 |
| GMI_ES01_c17486_304 | Ch_14D | 27.155 | KO_5_30 | 108 |
| GMI_ES02_c5098_397 | Ch_14D | 45.986 | KO_5_30 | 108 |
| GMI_ES15_c1710_467 | Ch_14D | 69.452 | KO_7_10_28 | 102 |
| GMI_DS_oPt-16568_206 | Ch_14D | 73.556 | KO_14 | 10.9 |
| GMI_DS_oPt-17047_370 | Ch_14D | 73.814 | KO_14 | 9.8 |
| GMI_ES01_c2878_533 | Ch_14D | 73.864 | KO_14 | 12.7 |
| GMI_ES02_c2109_540 | Ch_14D | 76.812 | KO_14 | 25.1 |
| GMI_ES17_c7023_741 | Ch_14D | 76.838 | KO_16_23 | 3.45 |
| GMI_ES17_c2251_283 | Ch_14D | 79.198 | KO_14 | 12.9 |
| GMI_ES01_c13432_100 | Ch_14D | 97.067 | KO_14 | 25.1 |
| GMI_ES17_c11262_129 | Ch_18D | 8.094 | KO_33 | 5.71 |
| GMI_ES17_c4863_294 | Ch_18D | 8.094 | KO_33 | 5.71 |
| GMI_ES02_c17401_684 | Ch_18D | 17.136 | KO_33 | 5.71 |
| GMI_ES01_c3759_358 | Ch_18D | 22.759 | KO_33 | 11 |
| GMI_ES01_c3919_84 | Ch_18D | 23.26 | KO_33 | 11 |
| GMI_ES15_c10388_464 | Ch_18D | 24.376 | KO_33 | 6.45 |
| GMI_ES02_c13817_311 | Ch_18D | 24.917 | KO_33 | 6.45 |
| GMI_ES02_c15540_620 | Ch_18D | 25.18 | KO_33 | 5.02 |
| GMI_ES02_c34493_237 | Ch_18D | 25.728 | KO_33 | 5.02 |
| GMI_ES01_lrc19995_311 | Ch_18D | 35.526 | KO_33 | 13.4 |
| GMI_ES17_c2976_706 | Ch_18D | 37.938 | KO_33 | 13.7 |
| GMI_ES02_c13415_701 | Ch_18D | 39.884 | KO_33 | 13.4 |
| GMI_DS_oPt-17923_173 | Ch_20D | 0 | KO_50 | 2.66 |
| GMI_DS_A3_39_99 | Ch_20D | 17.867 | KO_8 | 17.8 |
| GMI_ES17_lrc20004_472 | Ch_20D | 27.953 | KO_8 | 17.7 |
| GMI_ES02_c4144_303 | Ch_20D | 27.953 | KO_8 | 18.8 |
| GMI_ES01_lrc29552_212 | Ch_20D | 35.975 | KO_8 | 3.35 |
| GMI_ES15_c4217_214 | Ch_20D | 48.445 | KO_8 | 1.77 |
| GMI_ES15_c4017_310 | Ch_20D | 51.573 | KO_8 | 2.87 |
| GMI_ES15_c4463_508 | Ch_20D | 73.873 | KO_9 | 5.72 |
| GMI_DS_oPt-2653_332 | Ch_20D | 75.594 | KO_9 | 11 |
| GMI_ES01_c9159_455 | Ch_20D | 76.7 | KO_9 | 22.2 |
| GMI_ES01_c15405_389 | Ch_21D | 25.145 | KO_4_12_13 | 158 |
| GMI_DS_A3_468_401 | Ch_21D | 50.9 | KO_4_12_13 | 93.4 |
